# Genomic characterization between strains selected for death-feigning duration for avoiding attack of a beetle

**DOI:** 10.1038/s41598-021-00987-z

**Published:** 2021-11-08

**Authors:** Keisuke Tanaka, Ken Sasaki, Kentarou Matsumura, Shunsuke Yajima, Takahisa Miyatake

**Affiliations:** 1 NODAI Genome Research Center, Tokyo University of Agriculture, Tokyo, Japan; 2 Graduate School of Agriculture, Tamagawa University, Tokyo, Japan; 3 Graduate School of Agriculture, Kagawa University, Takamatsu, Japan; 4 Department of Bioscience, Tokyo University of Agriculture, Tokyo, Japan; 5 Graduate School of Environmental and Life Science, Okayama University, Okayama, Japan

**Keywords:** Behavioural ecology, Evolutionary ecology

## Abstract

Predator avoidance is an important behavior that affects the degree of adaptation of organisms. We compared the DNA variation of one of the predator-avoidance behaviors, the recently extensively studied "death-feigning behavior”, between the long strain bred for feigning death for a long time and the short strain bred for feigning death for a short time. To clarify how the difference in DNA sequences between the long and short strains corresponds to the physiological characteristics of the death-feigning duration at the transcriptome level, we performed comprehensive and comparative analyses of gene variants in *Tribolium castaneum* strains using DNA-resequencing. The duration of death feigning involves many gene pathways, including caffeine metabolism, tyrosine metabolism, tryptophan metabolism, metabolism of xenobiotics by cytochrome P450, longevity regulating pathways, and circadian rhythm. Artificial selection based on the duration of death feigning results in the preservation of variants of genes in these pathways in the long strain. This study suggests that many metabolic pathways and related genes may be involved in the decision-making process of anti-predator animal behavior by forming a network in addition to the tyrosine metabolic system, including dopamine, revealed in previous studies.

## Introduction

Since Edmunds^1^, much research has focused on the behaviors adopted by animals to avoid attack by enemies^2^. Death feigning (or thanatosis, tonic immobility, playing possum, playing dead, post-contact immobility, and so on) that have recently received special attention is one way to avoid enemy attack^3–6^. It has also been considered an adaptive behavior for females to avoid sexual cannibalism^7,8^, to avoid male harassment^9^ and for individuals to avoid worker aggressions in social insects^10^. Although the adaptive significance of death-feigning behavior has become widely recognized, very little research has been done on its molecular mechanisms.

Recently, Uchiyama et al.^11^ compared transcriptomes of beetle strains selected for short and long durations of death feigning. In *Tribolium castaneum*, strains divergently selected for short (S strains) and long (L strains) durations of death feigning, which is activated by external stimuli, have been established in the laboratory^3,12–14^.

A previous study identified 518 differentially expressed genes (DEGs) between the strains by transcriptome analysis, because RNA sequencing (RNA-seq), is rapidly gaining momentum in an effort to reveal the molecular mechanisms underlying physiological mechanisms^11^. The study revealed that tyrosine metabolic pathways including dopamine synthesis genes, stress-response genes, and insulin signaling pathways were differentially activated between individuals of short and long strains^11^. However, we cannot determine which part of the DNA caused the degree of transcription to change by transcriptome analysis alone.

A reciprocal crossing experiment between short and long strains for duration of death feigning showed that it occurred more frequently and for shorter periods in the F1 population with dominance in the short direction. From the F2 population, the death-feigning duration showed continuous segregation, indicating the duration of death feigning is controlled by polygenes in *T. castaneum*^15^. This result is consistent with the finding of many expressed RNAs involved by Uchiyama et al.^11^. The duration of death feigning has been found to be multilaterally expressed with other traits of insects: for example, locomotor activity in *T. castaneum*^12^, *T. confusum*^16^, and *T. freemani*^17^, flight ability in *Callosobruchus chinensis*^18^, life history traits in *C. chinensis*^19^, and mating behavior in *T. castaneum*^20^ and *C. chinensis*^21^. Therefore, it is easy to predict that the duration of a behavioral trait will be genetically affected by many other traits. Thus, we need to clarify how many genes influence the selection for the duration of death feigning on a DNA level.

To clarify how differences in DNA sequences between the long and short strains correspond to the physiological characteristics of the death-feigning duration at the transcriptome level, we performed comprehensive and comparative analyses of gene variants in *T. castaneum* strains using DNA-resequencing.

## Results

**Figure 1:**
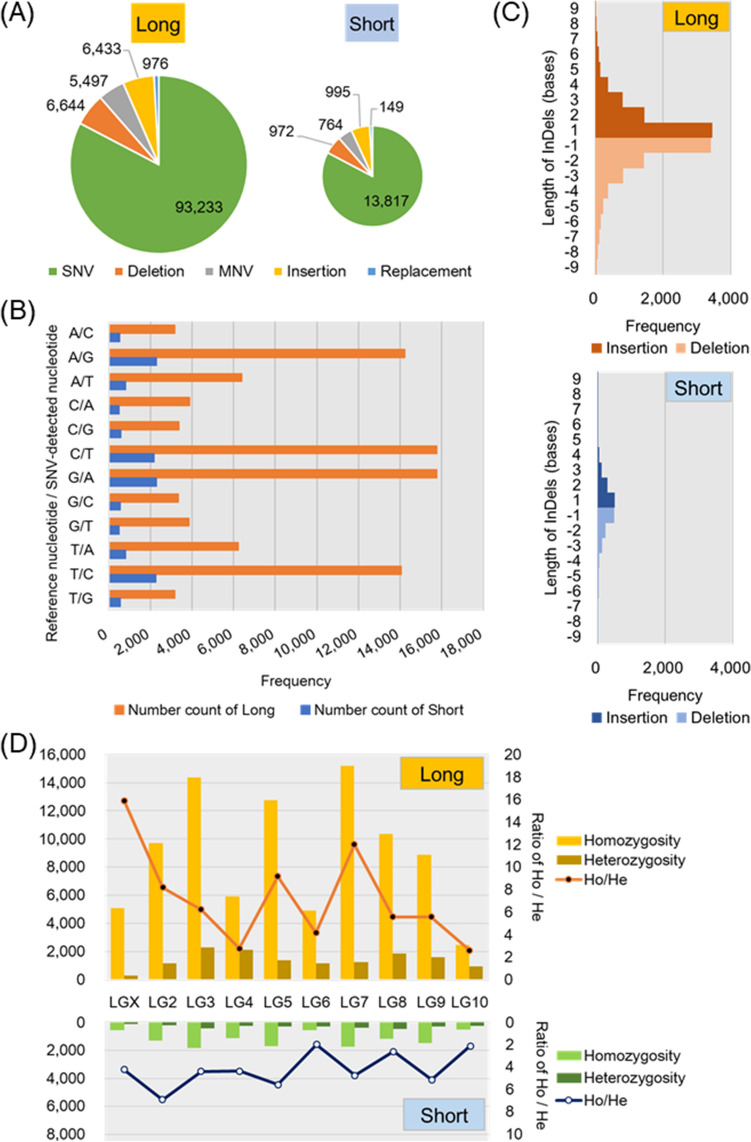
Analytical results of small variants of DNA sequence in a whole genome level in long and short strains. Proportion of small variants as SNV, MNV, deletion, insertion, and replacement in long and short strains (**A**). The numbers of small variants are indicated as the diameter of a pie graph. Frequencies of the SNVs in both long and short strains were compared with the reference nucleotide (**B**). Insertion and deletion ranged from one to nine bases in both long and short strains (**C**). Frequency of homozygosity or heterozygosity and its ratio in all linkage groups in long and short strains (**D**).

The present study compared DNA sequences in a whole genome between the long strain and standard genome samples as references or the short strain and standard ones in *T. castaneum*. The results of resequencing analysis showed variations of DNA sequence from the reference sequence in both long and short strains, and the variations were detected more frequently in the long strain in a whole genome. Small nucleotide variants (SNV), multi-nucleotide variants (MNV), deletion, insertion, and replacement were detected in a whole genome in long and short strains. The same DNA sequence variants sharing between long and short strains were removed for the analyses. The numbers of small variants in total were larger in long strains than short strains (Fig. 1, Tables S1 and S2). The most frequent type of small variants was SNV, and the proportions of SNV were 82.7% (93,233/112,783) in long strains and 82.8% (13,817/16,697) in short strains, respectively (Fig. 1A). The SNVs compared with the reference nucleotide occurred frequently between adenine and guanine or cytosine and thymine in both long and short strains (Fig. 1B), and the frequencies were up to three times as large as other base combinations, indicating more frequent transition and fewer transversion variants. Deletion and insertion ranged from one to nine bases in both long and short strains, with one base was frequently deleted or inserted (Fig. 1C). Homozygosity presented more frequently than heterozygosity in all linkage groups, but the rate of homozygosity to heterozygosity depended on the linkage groups (Fig. 1D). Homozygosity of variants was more frequent in linkage groups 3 (LG3), 5 (LG5) and 7 (LG7) than other linkage groups in both strains. The ratios of homozygosity to heterozygosity were the largest in LGX and LG2 in long and short strains, respectively.

**Figure 2:**
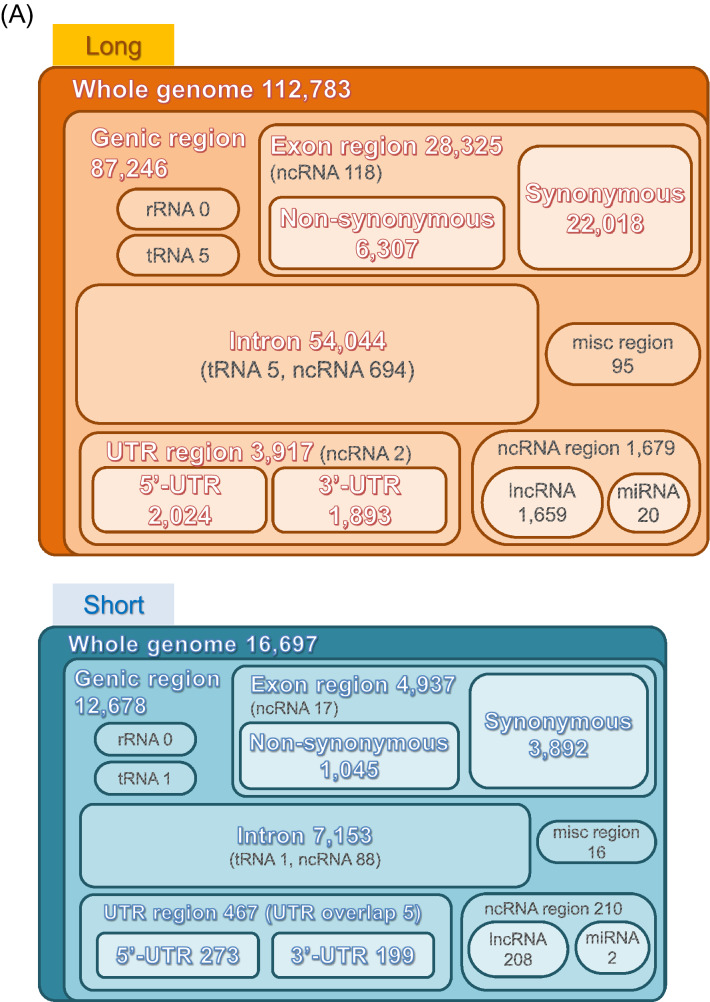

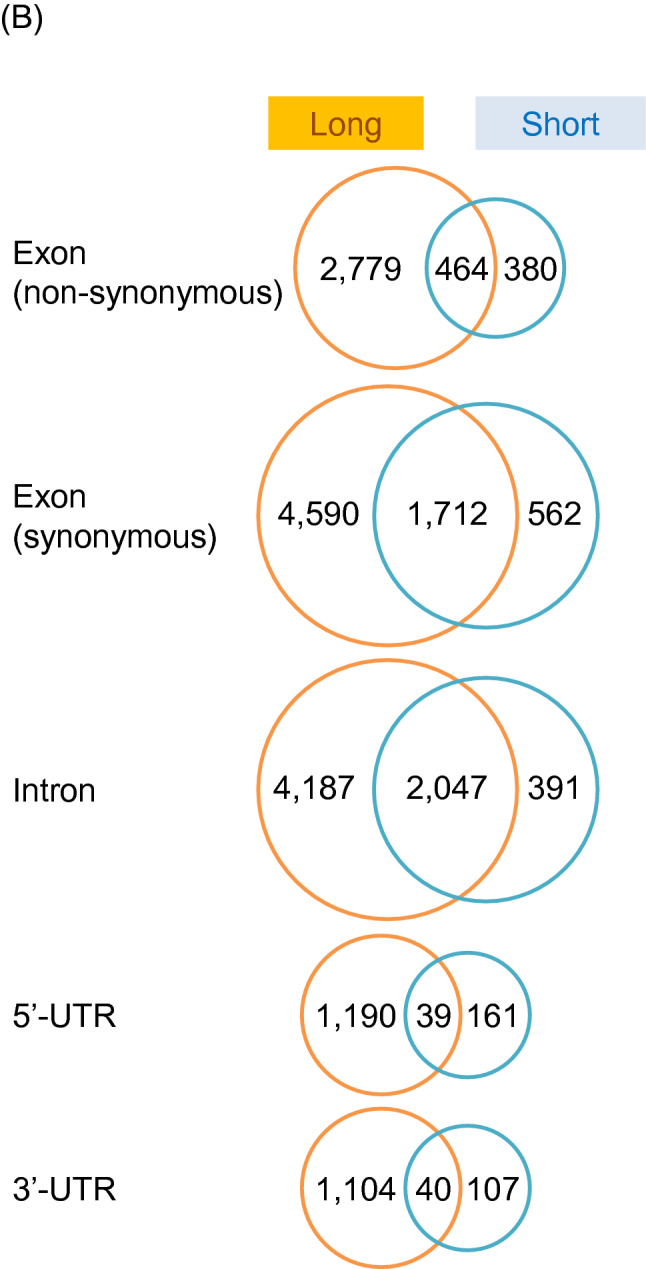

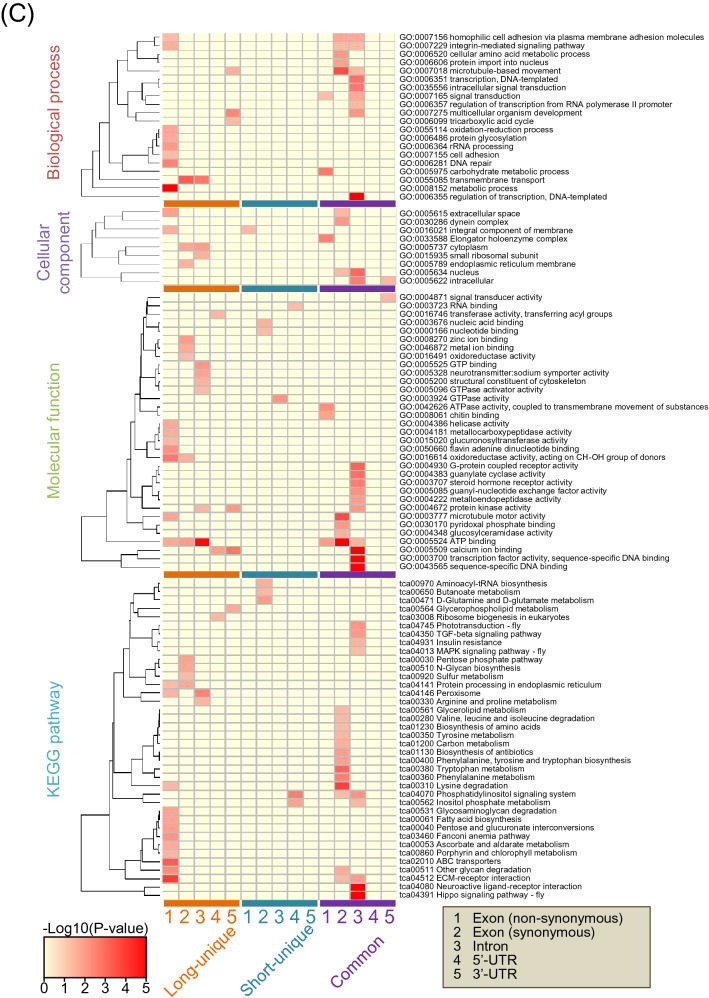
Analytical results of the position of small variants in a whole genome in long and short strains (**A**) Numbers of variants in genic region including exon region, intron, UTR and other non-cording regions were indicated. As shown in parentheses, some ncRNAs and tRNAs were contained in exon, intron, and UTR regions. In short strain, there were five regions where two different genes overlap in 5′-UTR and 3′-UTR, respectively. Numbers of genes with variants in exon, intron and UTR regions in long and short strains (**B**). Numbers of long-unique, short-unique and common genes were shown by Venn diagrams. Common genes contain variants with different DNA sequences between long and short strains. Enrichment analyses of the function of genes with variants sorted into four categories (biological process, cellular component, molecular function, and KEGG pathway) (**C**). The heatmap is generated using the R package “gplots” (version 3.1.1, https://cran.r-project.org/web/packages/gplots/index.html). The list of each ontology shows the ID and term. The KO id is shown by a three- or four-letter organism code, the first-letter of the genus name and the first two- or three-letters of the species name of the scientific name of the organism, with pathway number. For example, Neuroactive ligand-receptor interaction of *Tribolium castaneum* is shown as "tca04080".

The variants distributed in cording and non-cording regions. Figure 2A shows the results of narrowing down the variants in genic region from the variants in a whole genome in the long and short strains, and then aggregating the variants information in the exon, intron, URT and other regions. In all genic region, numbers of variants were larger in long strain than short strain. Then, genes containing these variants were counted in each strain (Fig. 2B). In exon region, genes with nonsynonymous variants were more numerous in the long strain (3243) than the short strain (844), and 464 common genes containing different DNA sequence variants between the strains were detected (Fig. 2B). In the genes with synonymous variants or the genes with variants in intron or UTR, the numbers of genes in long strain were constantly larger than those in short strain (Fig. 2B). The functions of long-unique, short-unique and common genes with variants were sorted into four categories by enrichment analyses as gene ontology (GO) and Kyoto Encyclopedia of Genes and Genomes (KEGG) ongoloty (KO) terms (Fig. 2C, Table S3). In the biological process, cellular component, and molecular function, and KEGG pathway, characteristics of nonsynonymous variants in long-unique, short-unique and common genes did not basically overlap among them, indicating specific selection of gene characteristics for each strain. Characteristics of synonymous variants were also sorted, but the synonymous variants may not influence the amino acid sequence of the gene and structure of the protein translated, rather these characteristics may be necessary to maintain the strain and preserved under artificial selection. Variants in intron and UTR may have potential effects on the gene expression, but should be investigated in detail in future study. Analyses of cis-regulatory elements might be important to understand regulation of gene expression, but the information on this region in *T. castaneum* is not available, therefore, the variants in cis-regulatory elements could not be analyzed.

**Figure 3:**
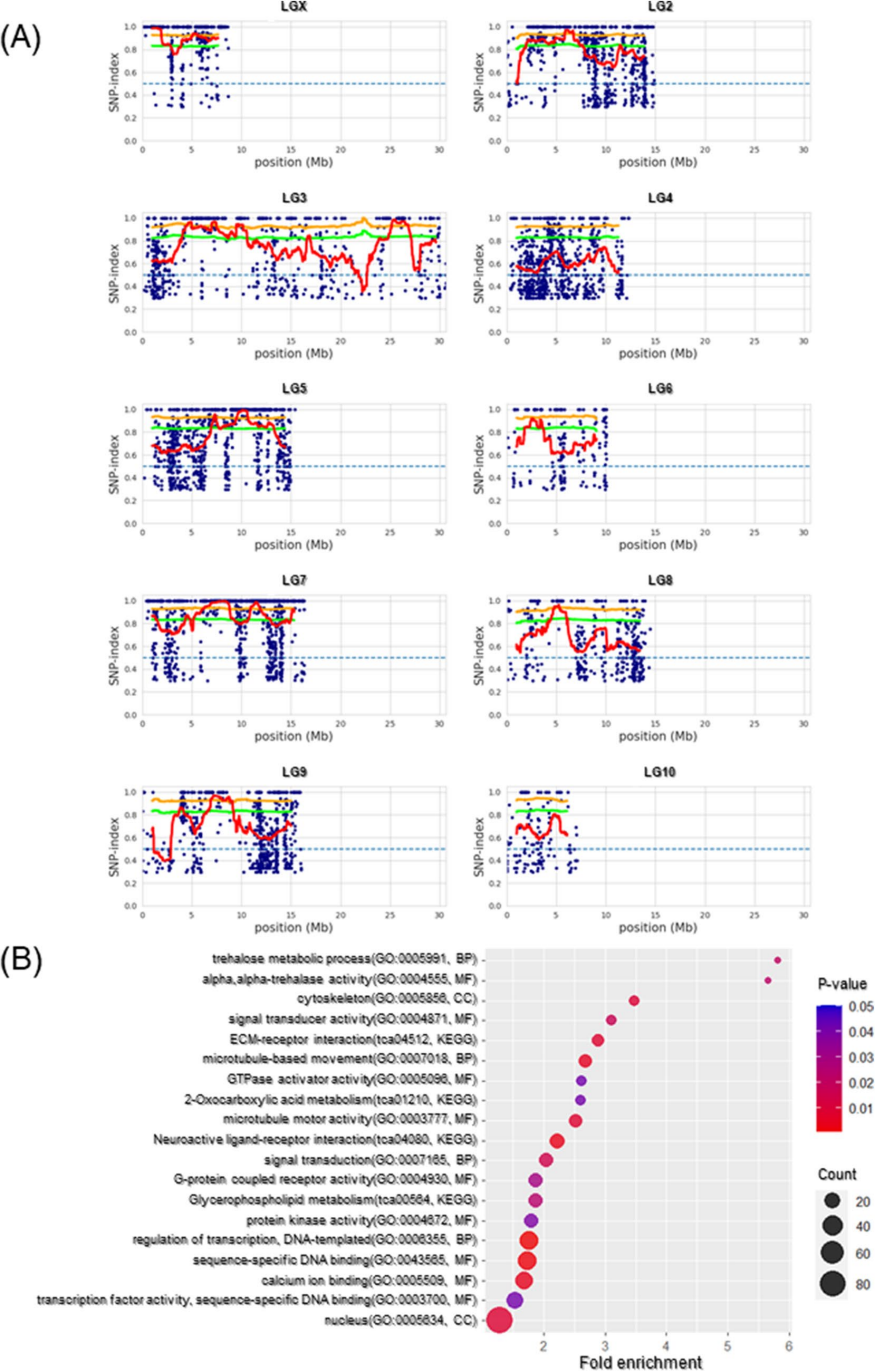
Analytical results of MutMap approach in each linkage group (**A**) and enrichment analysis (**B**). In (**A**), the red line indicates an approximate line of plot data. The green and orange threshold lines indicate 95% and 99% significance, respectively. The dot plot in (**B**) is generated using the R package “ggplot” (version 3.3.5, https://cran.r-project.org/web/packages/ggplot2/index.html).

To explore the position of genes with variants associated with duration of death feigning in linkage groups, bulk segregant analysis was carried out (Fig. 3). The red approximate lines of the plot data crossed over the green threshold lines (*P* < 0.05) in linkage groups X, 2, 3, 5, 6, 7, 8, and 9, but did not linkage groups 4 and 10 (Fig. 3A, Table S4). Further, the approximate lines in linkage groups X, 2, 3, 5, 7, 8, and 9, crossed over the orange threshold lines (*P* < 0.01), but those in linkage group 6 did not. Therefore, the genes in linkage groups X, 2, 3, 5, 7, 8, and 9 were candidates selected artificially on the basis of the duration of death feigning. Enrichment analyses of the gene characteristics extracted KO and GO terms including “neuroactive ligand-receptor interaction” and “G-protein coupled receptor activity”, these terms seem to be associated with monoamine receptor activity (Fig. 3B, Table S5).

**Figure 4:**
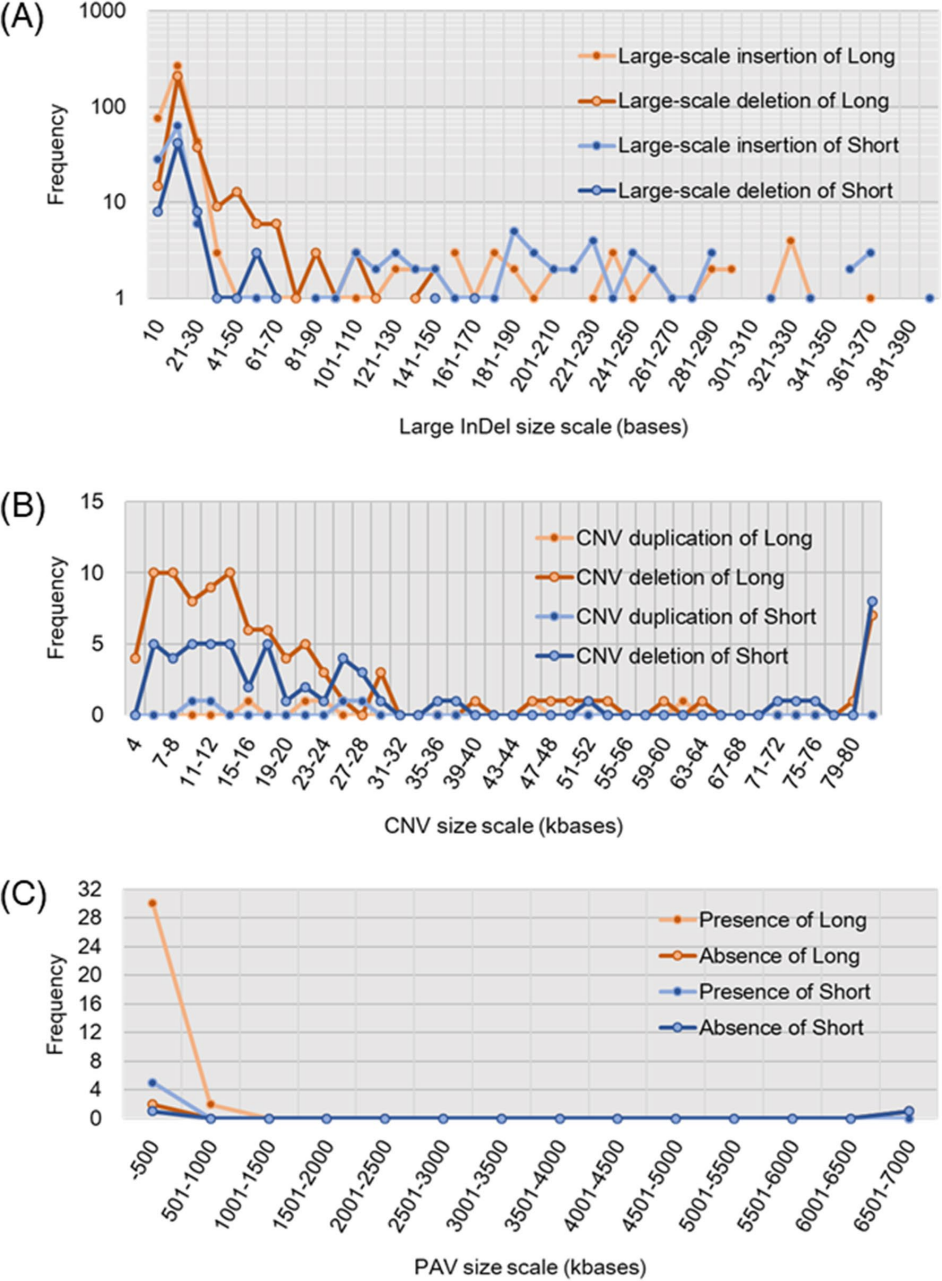
Structural variations including large-scale insertion and deletion (InDel) in a whole genome (**A**), copy number variation (CNV) (**B**), and presence/absence variation (PAV) (**C**) in long and short strains. Large-scale InDels were shown diagrammatically from 10 to 390 bases. CNV duplication and deletions were shown diagrammatically from 4 to 80 kbases. PAV were shown diagrammatically from 0 to 7000 kbases.

Structural variations including large-scale insertion and deletion (InDel), copy number variation (CNV), and presence/absence variation (PAV) were analyzed in a whole genome level in both strains (Fig. 4, Tables S6–S11). Large-scale insertions and deletions were analyzed in 10–393 bases, and 11–20 bases of insertion and deletion were the most frequent in both strains (Fig. 4A). CNV deletions were present more frequently in sizes ranging from 5 to 14 kbases than in other size scales that we examined in both strains, whereas CNV duplications were constantly less frequent at 0 or 1 cases (Fig. 4B). In a larger size scale of nucleotides, up to 7000 kbases, the presence of variations less than 500 kbases of nucleotide sizes was most frequent in the long strain (Fig. 4C).

**Figure 5:**
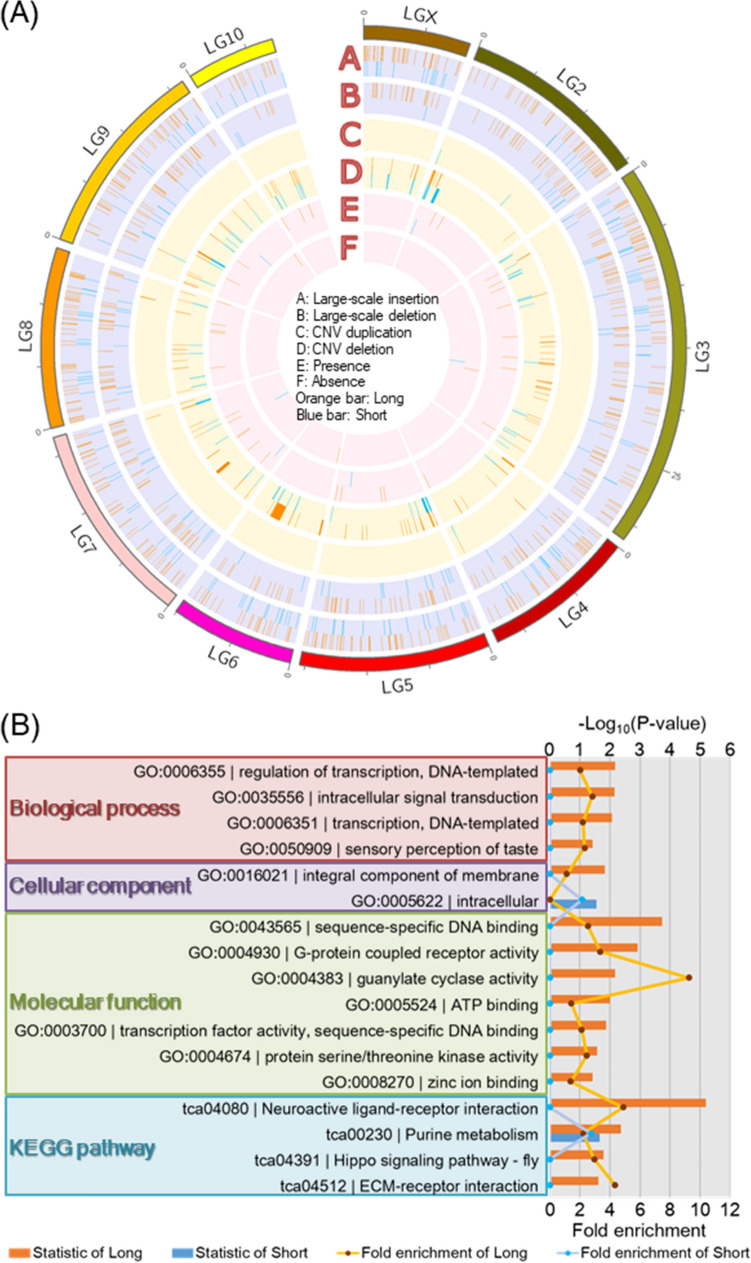
Structural variations on each linkage group in long and short strains (**A**). The circos plot is drawn using Circos (version 0.69-9, http://circos.ca/). Structural variations include large-scale insertion and deletion, CNV duplication and deletion, and presence and absence of variation. The long and short strains are indicated by orange and blue lines, respectively. GO and KO terms are from function of genes with variants (**B**).

All of these are illustrated on each linkage group in Fig. 5A, indicating large-scale insertions and deletions constantly appearing in each linkage group (A and B), less frequent CNV duplications (C), and more frequent CNV deletions (D). Large CNV deletions were present in LG6 and LG7 in the long strain and in LG2 in the short strain, respectively. These variations were sorted into GO and KO terms (Fig. 5B, Table S12). The term of “neuroactive ligand-receptor interaction” had the largest statistical value in the long strain.

**Figure 6:**
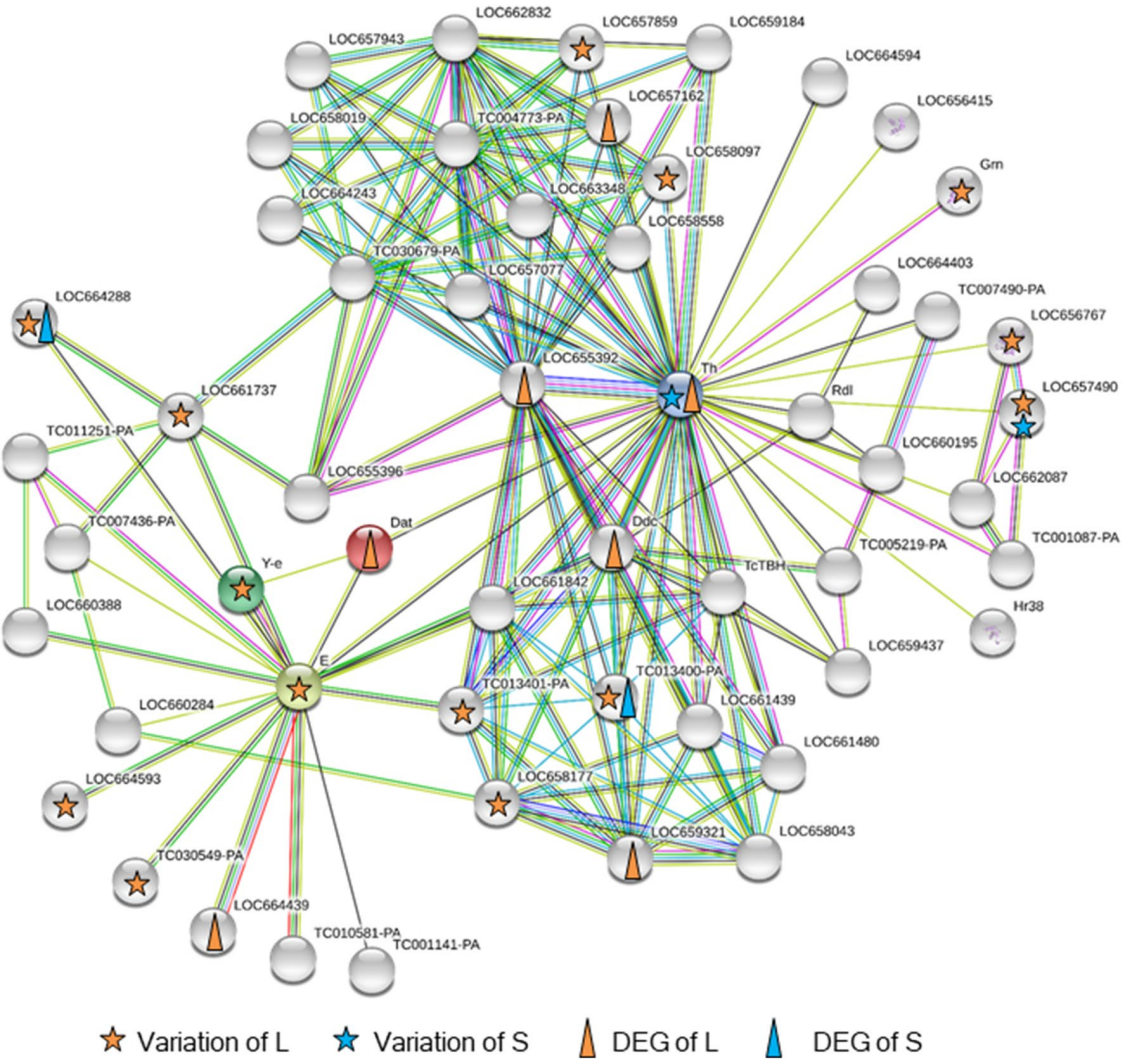
Protein–protein interaction (PPI) network including enzymes involved in dopamine metabolism. Lines indicate the relationships between genes. The network map is drawn using the STRING (version 11.0, https://string-db.org/). Stars and triangles indicate genes with nonsynonymous variants and differentially expressed genes (DEGs), respectively.

A protein–protein interaction (PPI) network including enzymes involved in dopamine metabolism was constructed (Fig. 6). Tyrosine hydroxylase (*Th*) was connected with DOPA decarboxylase (*Ddc*) and dopamine *N*-acetyltransferase (*Dat*), and these enzymes have been reported as differentially expressed genes in the long strain analyzed by RNA-seq^13^. *Th* also had variations of DNA sequence (nonsynonymous variants) in the short strain (Fig. 6). Among the PPI network, proteins with nonsynonymous variants were more frequent in the long strain. Yellow-like protein had nonsynonymous variants in the long strain, and it was indirectly connected with *Ddc* and *Th* and directly with *Dat*.

**Figure 7:**
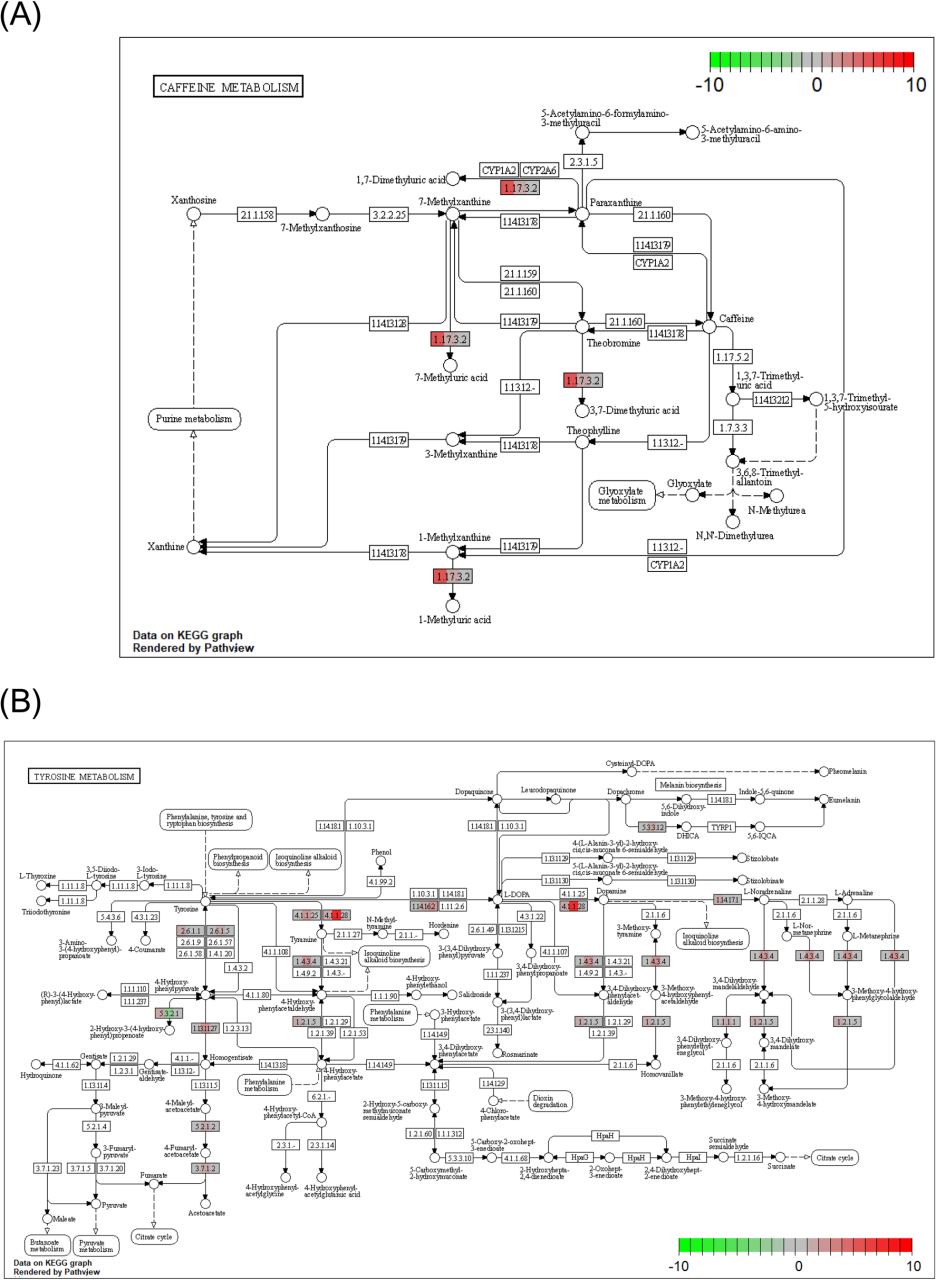

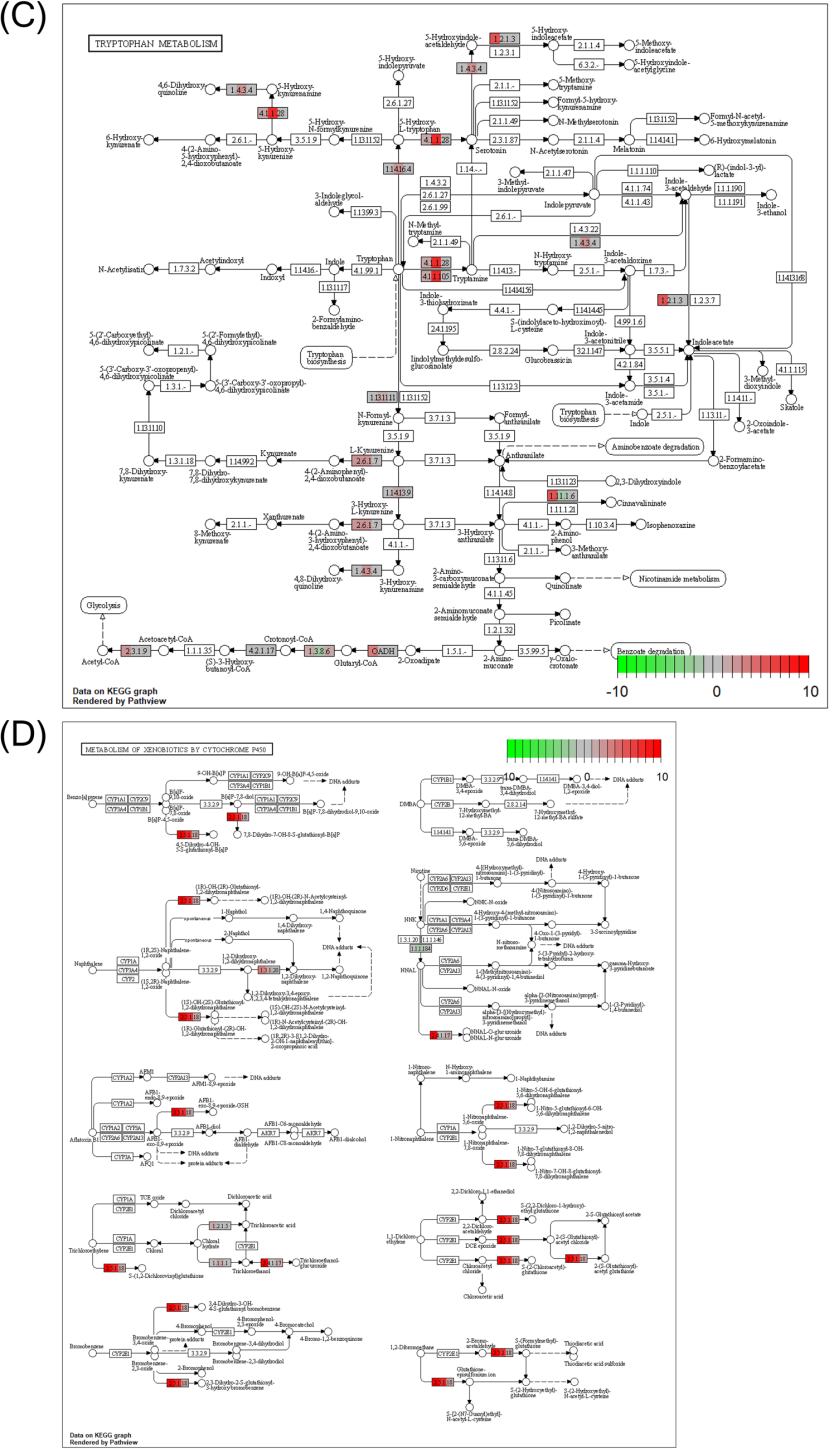

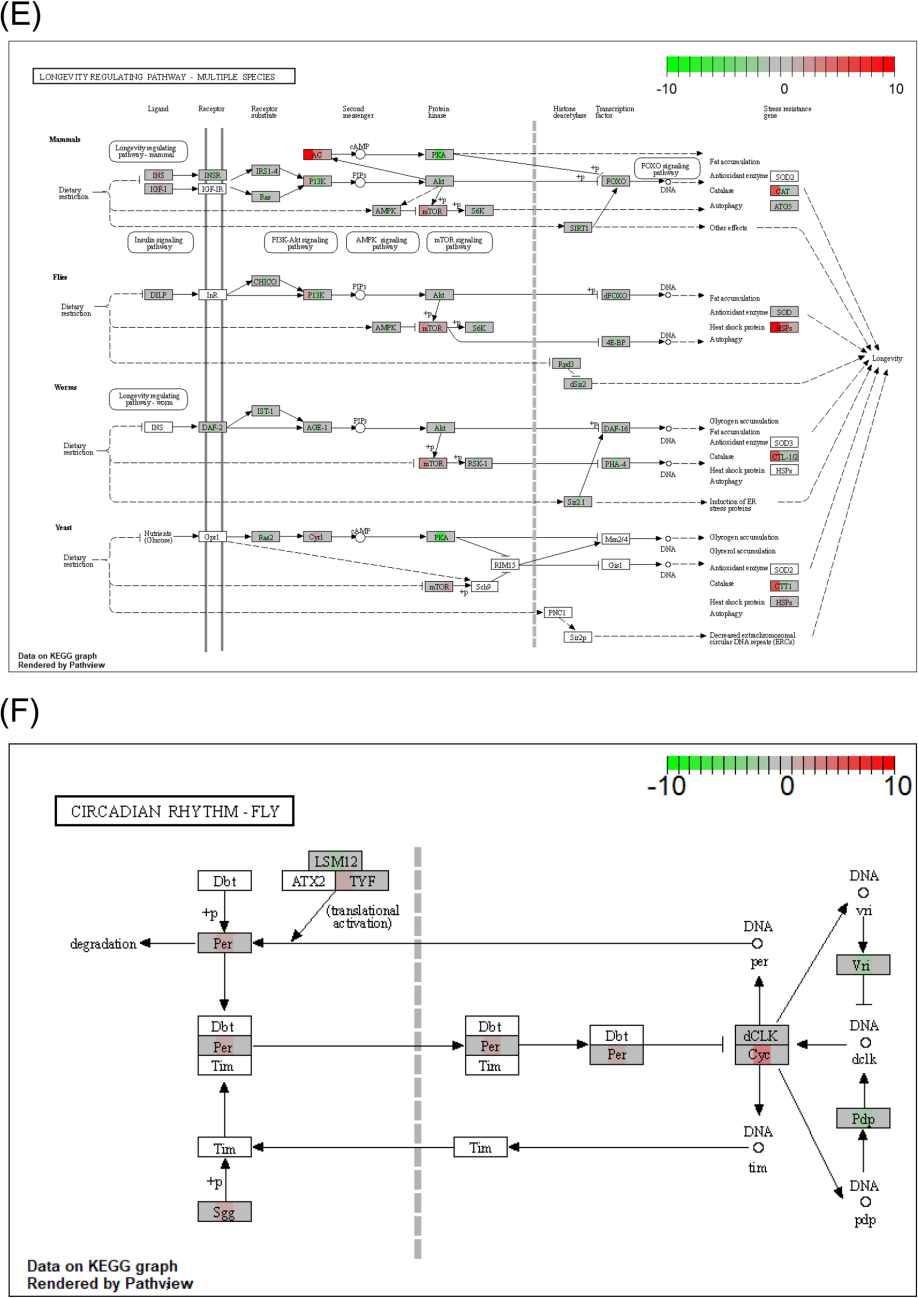
Functional genes with frequency of nonsynonymous variants in KEGG pathways. Each pathway map is generated using the R package “Pathview” (version 1.30.1, https://bioconductor.org/packages/release/bioc/html/pathview.html). Caffeine metabolism (**A**), tyrosine metabolism (**B**), tryptophan metabolism (**C**), metabolism of xenobiotics by cytochrome P450 (**D**), longevity regulating pathway (**E**) and circadian rhythm (**F**) are indicated. Reference nucleotide (Tcas5.2) obtained from the NCBI genome database. The color gradient in the rectangular box represents nonsynonymous variant burden or differences in the intensity of fold change by RNA-seq between the long and short strains (left, long variant burden; center, fold change; right, short variant burden). The upper fold change means the expression value of the long strain is higher than that of the short strain. The lower fold change means the opposite.

Pathways containing genes with nonsynonymous variants in both long and short strains were analyzed in “caffeine metabolism (tca00232)” (Fig. 7A), “tyrosine metabolism (tca00350)” (Fig. 7B), “tryptophan metabolism (tca00380)” (Fig. 7C), “metabolism of xenobiotics by cytochrome P450 (tca00980)” (Fig. 7D), “longevity regulating pathway—multiple species (tca04213)” (Fig. 7E), and “circadian rhythm—fly (tca04711)” (Fig. 7F). Tyrosine metabolism and longevity-regulating pathways have been listed as pathways containing focal genes with different expressions between long and short strains as detected by RNA-seq^13^. The numbers of variants of genes in these pathways were larger in the long strain than the short strain (Fig. 7).

## Discussion

We compared DNA sequences between the long strain and standard genome samples, or the short strain and standard ones. This is because a reliable DNA sequences of standard genome samples. Since the variants may contain biased sequences by experimental isolation from local population, the same DNA sequence variants sharing between long and short strains were removed for the analyses. We must note that in considering the following discussion and conclusions, we should pay attention to the bottleneck of the strains used in the experiment, because the strains analyzed have been artificially selected for more than 15 generations^12^.

Even variants contain biased sequences via bottleneck effects, number of variants and number of genes containing variants in a whole genome were largely different between long and short strains, and much larger in long strain than short one. These results suggest that many variants in long strain were selected artificially based on the duration of death feigning. Characteristics of long-unique, short-unique and common genes containing variants did not basically overlap among them, suggesting specific selection of gene characteristics in each strain and preservation of important gene characteristics. The specific selection of gene characteristics was also suggested by the results of a protein–protein interaction network including enzymes involved in dopamine metabolism. The results indicated that several enzyme proteins have only variation of long strain. While, the other enzyme proteins with variation of both long and short strains may indicate biased DNA sequence by experimental isolation via bottleneck effect, or key enzymes to adjust biosynthesis toward positive or negative for each strain.

The analyses detected small variants, including SNV, MNV, deletion, insertion and replacement in a whole genome level in both long and short strains. Especially, the long strain had more variants in a particular gene than those in the short strain. This was the case in the structural variations including large-scale InDel, CNV, and PAV. These large-scale variations were frequently detected in the long strain. The frequent variations of DNA sequences in the long strain may cause production of more diverse gene expressions in the long strain. Artificial selection based on the duration of death feigning results in the preservation of variants in genes in these particular metabolic and signaling pathways in the long strain, whereas the short strain with fewer variants of the DNA sequence might be selected more strongly than the long strain. Why were there larger variations in several genomic regions in the long line than in the short line? For example, the fact that there are more variants in the long strains in general seems to be related to the asymmetric response of selection to the duration of pseudo-death, as shown by Miyatake et al.^3^. In fact, long strains have a larger direct response to selection on death-feigning duration than short strains^3,12,14^.

### Caffeine metabolism

In the KEGG pathway, there were significant inter-strain differences in CYOIA2, 7-methyluric acids, 3,7-dimethyluric acid, and 1-methyluric acid (Fig. 7A). Oral ingestion and injection of caffeine have been shown to shorten the death-feigning duration of adults in the long strains of *T. castaneum*^22^. As similar phenomenon has been found in the related species *T. confusum*, in which strains selected for longer duration had shorter death-feigning duration when they had ingested caffeine orally^23^. Nakayama et al.^23^ concluded that the dopaminergic system plays an important part in controlling the genetic correlation between death feigning and activity levels in these *Tribolium* species. Caffeine is known as a dopamine activator. Therefore, the present result demonstrates the relationship between the caffeine-dopamine system and death feigning at the genome level.

### Tyrosine metabolism

The results of the transcriptome by the RNA-seq study^11^ and the current DNA analysis are consistent with the tyrosine metabolic system (Fig. 7B). Variants in DNA sequence were found in four genes in the long strain and one gene with variants in two sequence locations in the short strain. One of the four genes in the long strain was “*Ddc*”, which is involved in the direct synthesis of dopamine, and one of the genes in the short strain was “*Th*”, which is involved in the synthesis of L-dopa, the precursor of dopamine. In the previous RNA-seq study^11^, the expression levels of these genes were found to be significantly higher in the long strain, so they may be affected by the DNA sequences revealed in the present results. The tyrosine and phenylalanine metabolic systems might be associated with the tryptophan metabolic system. Genome analysis showed more variants in genes of the tryptophan metabolic system than in the tyrosine metabolic system, but the proteins of the tryptophan metabolic system are related to the tyrosine metabolism. Because many variants seem to occur in the gene region related to glucose metabolism, the difference in the amount of ATP produced as a result of glucose metabolism may also affect the effects of caffeine.

### Tryptophan metabolism

In the KEGG pathway of tryptophan metabolism, 16 genes showed differences between strains (Fig. 7C). There are four possible explanations for how mutations in the tryptophan metabolic system indirectly affect gene expression. (1) Differences in serotonin levels and serotonin synthase gene expression may be related to the tryptophan metabolism. (2) The amount of melatonin, a metabolite of serotonin, and together with serotonin, is related to variations in circadian rhythms (also see Fig. 7F), which will be discussed later. (3) Metabolism of the ommochrome system (ommochrome is a pigment substance that produces the color of the compound eye (individual eye)), and may be related to this metabolic system in relation to vision. (4) Synthesis of tryptophan into proteins that are needed (tryptophan is an amino acid, so it is a raw material for proteins). To prove these hypotheses, we need to conduct further studies at physiological and/or molecular levels.

### Metabolism of xenobiotics by cytochrome P450

Eight genes differed between strains (see Fig. 7D). At phenotypic level, comparisons of metabolisms between the strains have not been conducted in *T. castaneum*. On the other hand, in *Callosobruchus chinensis*, long-lines selected for longer duration of death feigning exhibited higher rates of emergence, laid bigger eggs compared with strains selected for shorter duration of death feigning and greater reproductive effort, and also had a tendency to develop faster^19^. These changes in life-history traits, correlated with the duration of death feigning, may be related to metabolism in insects because strains with longer duration of death feigning can conserve energy to reproduce during their lives. This trade-off between anti-predator strategies and energy conservation might relate to the metabolism. Therefore, it is required to measure differences between the metabolisms of long and short strains of *T. castaneum*.

### Longevity regulating pathway

Differences in expression in the longevity regulating pathway were also found in an RNA-seq study^13^ (Fig. 7E). This pathway contains a catalase gene (*CAT*) seen in a larger number of variants in the long strain. Kiyotake et al.^24^ has reported that the longer strains had lower relative expressions of *CAT*. This lower expression of *CAT* might be involved in the preservation of a large number of *CAT* variants in the long strain. Because *Tribolium* species have long longevity (more than 200 days), no comparison of lifetimes between strains was made. On the other hand, in *Callosobruchus chinensis*, long strains selected for longer duration of death feigning exhibited greater longevity compared short strains selected for shorter duration of death feigning and greater reproductive effort, and also had a tendency toward faster development^19^.

### Circadian rhythm

Long strain beetles have a significantly larger variation than short strain beetles in the three circadian genes including *Per* (period), *Sgg* (Shaggy), and *Cyc* (Cycle). Although the previous RNA-seq study showed the expression of *Dbt* (doubletime) is only slightly different between the short and long strains^11^, the present DNA re-sequence did not find nonsynonymous variation in the *Dbt* gene between the strains (Fig. 7F). Therefore, it will be very interesting to compare the circadian rhythm of beetles derived from long and short strains in the future.

### Other genes

There are other differences in the DNA sequences of the long and short strains; for example, the glutathione metabolism pathway and stress-resistance and heat-shock genes. Using the same strains as the present study, Kiyotake et al.^26^ showed that the longer strains had lower relative expression of catalase (*CAT*) and growth-blocking peptide (*GBP*) genes compared to the short strains. These genes are related to anti-stress capacity, and Kiyotake et al.^24^ showed that the long-strain beetles are significantly more sensitive to environmental stressors such as mechanical vibration and high or low temperatures than the short-strain beetles. The difference in stress resistance between the strains may relate to stress-resistance genes and the heat-shock system.

## Conclusion

The duration of death feigning is related to many gene pathways, including caffeine metabolism, tyrosine metabolism, tryptophan metabolism, metabolism of xenobiotics by cytochrome P450, longevity regulating pathway, and circadian rhythm. Artificial selection based on the duration of death feigning results in the preservation of variants in genes in these pathways in the long strain. An animal's decision on when to wake up from a near-death experience is closely related to its success in avoiding predation^5,25,26^. This study suggests that many metabolic pathways and related genes may be involved in the decision-making process of anti-predator animal behavior by forming a network in addition to the tyrosine metabolic system including dopamine revealed in previous studies.

## Materials and methods

### Insects

The red flour beetle, *Tribolium castaneum* (Herbst 1797), is a stored-product insect found worldwide and a model genome species, designated by the *Tribolium* Genome Sequencing Consortium^27^. The protocol for artificial selection for the duration of death feigning was described in Miyatake et al.^3^ Briefly, the duration of death feigning was measured in 100 male and 100 female adult beetles that were randomly selected. From these populations, ten males and ten females with the shortest and longest durations of death feigning were allowed to reproduce for the next generation. The selection regime was continued for more than 20 generations^12–14^.

### DNA extraction

Female individuals in short or long strains were frozen by liquid nitrogen. Head and thoracic tissues without legs were removed from the frozen bodies by a pair of fine spring scissors. Each tissue was homogenized by the scissors and an electric homogenizer (T10 + S10N-5G, IKA Works, Staufen, Germany) in an extraction buffer from an ISOGEN kit (Nippongene, Tokyo, Japan) according to the manufacturer’s instructions. The quality and quantity of the extracted DNA were determined at 230, 260, and 280 nm using a spectrophotometer (NanodropTM 2000, Thermo Fisher Scientific, MA, USA).

### Library construction and sequencing

A total of 1–10 ng genomic DNA was fragmented by shearing to an average fragment size of 300 bp using an Adaptive Focused Acoustics sonicator (Covaris, Woburn, MA, USA). After purification, the paired-end DNA library was constructed using a KAPA Hyper Prep kit (KAPA Biosystems, Wilmington, MA, USA). The fragmented DNA was end-repaired, dA-tailed, and ligated with the paired-end adapter according to the manufacturer’s instructions. The adapter-ligated DNA was amplified by 14 cycles of high-fidelity polymerase chain reaction (PCR) amplification. Library quality and concentration were assessed using an Agilent Bioanalyzer 2100 (Agilent Technologies, Waldbronn, Germany) and an Agilent DNA 1000 kit. In addition, the library concentration was precisely determined using a KAPA Library Quantification Kit (Kapa Biosystems).

The paired-end libraries were sequenced by 200 cycles (2 × 100 bp) using the HiSeq 2500 (Illumina, San Diego, CA, USA). Reads were generated in FASTQ format using the conversion software bcl2fastq2 (Illumina, version 2.18, https://jp.support.illumina.com/sequencing/sequencing_software/bcl2fastq-conversion-software/downloads.html). We submitted the read data to the Read Archive of DDBJ (accession number DRA011837).

### Read mapping to reference whole genome

A series of data analyses was processed using CLC Genomics Workbench 12 (Qiagen, Hilden, Germany). After adapter trimming and quality filtering, the clean read data were mapped to the reference genome of *T. castaneum* (Tcas5.2) that was obtained from the NCBI genome database (https://www.ncbi.nlm/nih.gov/). The mapping parameters were as follows: mismatch cost = 2, insertion cost = 3, deletion cost = 3, length fraction = 0.9, and similarity fraction = 0.9. After local realignment of the mapped reads, duplicate PCR reads were discarded.

### Small variant detection

Variant calling based on single nucleotide variation (SNV) and insertion and deletion (InDel) was performed using the CLC Genomics Workbench built-in tool “Fixed Ploidy Variant Detection”. The calling parameters were as follows: ploidy = 2, required variant probability = 90.0, minimum coverage = 10, minimum frequency = 20, minimum central quality = 40, and minimum neighborhood quality = 30. Furthermore, high-quality variants were selected using QUAL = 80. Identical variations sharing between the long and short strains was excluded to take no thought of the standing variations in the original population. Variant genes contained in for each exon (nonsynonymous or synonymous), intron, 5′-UTR, and 3′-UTR were enriched as Gene Ontology (GO) and Kyoto Encyclopedia of Genes and Genomes (KEGG) ontology (KO) terms using the web-based tool “DAVID 6.8” (https://david.ncifcrf.gov)^28^. The enriched terms were statistically analyzed using the modified Fisher’s exact test (*p* < 0.05) contained in the tool.

### Bulk segregant analysis

Bulked read data consisting seven individuals in each strain were prepared to detect population-level variation and analyzed using MutMap pipeline (version 2.3.2, https://github.com/YuSugihara/MutMap)^28^. The SNP-index calculation parameters were as follows: window size = 2000 kb, step size = 100 kb. Significant loci were selected by upper side than the surrounding regions at 95% confidence interval. The genes contained therein were picked up and enriched as GO and KO terms using the web-based tool “DAVID 6.8”. The enriched terms were statistically analyzed using the modified Fisher’s exact test (*P* < 0.05) contained in the tool.

### Structural variant detection

Variant calling based on copy number variation (CNV) was performed using CNVnator (version 0.3.3, https://github.com/abyzovlab/CNVnator)^29^. The calling parameters were as follows: size ≥ 4000, normalized RD ≥ 2 or ≤ 0.5, and E-value by *t*-test statistics < 0.05. In comparison, variant calling based on presence/absence variation (PAV) was run using the CLC Genomics Workbench built-in tool “InDel and Structural Variants”. Identical variations sharing between the long and short strains was excluded for the same thought as the small variant detection. The genes contained in the identified regions were enriched as GO and KO terms using the web-based tool “DAVID 6.8”^30^. The enriched terms were statistically analyzed using the modified Fisher’s exact test (*p* < 0.05) contained in the tool.

### Functional annotation between nonsynonymous variants and differentially expressed genes

We investigated whether resequencing data was relevant to previous transcriptome analysis data^11^. A protein–protein interaction (PPI) network was constructed using the STRING (version 11.0, https://string-db.org/)^31^. The target protein searched for DAT having a central role in this study. The parameters were customized as follows for the output data: minimum required interaction score, 0.600; max number of interactors to show no more than 50 interactors (both 1st and 2nd shells). Pathway analysis was performed for “caffeine metabolism (tca00232)”, “tyrosine metabolism (tca00350)”, “tryptophan metabolism (tca00380)”, “metabolism of xenobiotics by cytochrome P450 (tca00980)”, “longevity regulating pathway—multiple species (tca04213)”, and “circadian rhythm—fly (tca04711)” using the R package “Pathview” (version 1.30.1, https://bioconductor.org/packages/release/bioc/html/pathview.html)^32^.

### Ethical approval and informed consent

N/A because of insects.

## Supplementary Information

Supplementary Tables.

## Data Availability

The datasets generated during and/or analyzed during the current study are available from the corresponding author on reasonable request.
