# Publisher Correction: Genomic characterization between strains selected for death-feigning duration for avoiding attack of a beetle

**DOI:** 10.1038/s41598-021-02478-7

**Published:** 2021-11-22

**Authors:** Keisuke Tanaka, Ken Sasaki, Kentarou Matsumura, Shunsuke Yajima, Takahisa Miyatake

**Affiliations:** 1 NODAI Genome Research Center, Tokyo University of Agriculture, Tokyo, Japan; 2 Graduate School of Agriculture, Tamagawa University, Tokyo, Japan; 3 Graduate School of Agriculture, Kagawa University, Takamatsu, Japan; 4 Department of Bioscience, Tokyo University of Agriculture, Tokyo, Japan; 5 Graduate School of Environmental and Life Science, Okayama University, Okayama, Japan

Correction to: *Scientific Reports* 10.1038/s41598-021-00987-z, published online 08 November 2021

The Funding section in the original version of this Article was omitted. The Funding section now reads:

“This work was supported by the Japan Society for the Promotion of Science Grant-in-Aid for Scientific Research Grants 18H02510 and 21H02568 (to T.M., K.S.), and the Cooperative Research Grant of the Genome Research for BioResource, NODAI Genome Research Center, Tokyo University of Agriculture (to T.M., K.S., S.Y.).”

The original Article has been corrected.

